# Fluid balance in critically ill children with lower respiratory tract viral infection: a cohort study

**DOI:** 10.1186/s44158-023-00093-8

**Published:** 2023-04-28

**Authors:** Chiara Robino, Guido Toncelli, Laura Arianna Sorrentino, Antonio Fioccola, Brigida Tedesco, Cristina Giugni, Manuela L’Erario, Zaccaria Ricci

**Affiliations:** 1 Department of Anesthesia and Critical Care, Pediatric Intensive Care Unit, Meyer Children’s University Hospital, IRCCS, Florence, Italy; 2 Department of Health Sciences, Section of Anesthesiology and Intensive Care, University of Florence, Florence, Italy

**Keywords:** Bronchiolitis, Fluid balance, Fluid overload, Respiratory support

## Abstract

**Background:**

Increasing evidence has associated positive fluid balance of critically ill patients with poor outcomes. The aim of this study was to explore the pattern of daily fluid balances and their association with outcomes in critically ill children with lower respiratory tract viral infection.

**Methods:**

A retrospective single-center study was conducted, in children supported with high-flow nasal cannula, non-invasive ventilation, or invasive ventilation. Median (interquartile range) daily fluid balances, cumulative fluid overload (FO) and peak FO variation, indexed as the % of admission body weight, over the first week of Pediatric Intensive Care Unit admission, and their association with the duration of respiratory support were assessed.

**Results:**

Overall, 94 patients with a median age of 6.9 (1.9–18) months, and a respiratory support duration of 4 (2–7) days, showed a median (interquartile range) daily fluid balance of 18 (4.5–19.5) ml/kg at day 1, which decreased up to day 3 to 5.9 (− 14 to 24.9) ml/kg and increased to 13 (− 11 to 29.9) ml/kg at day 7 (*p* = 0.001). Median cumulative FO% was 4.6 (− 0.8 to 11) and peak FO% was 5.7 (1.9–12.4). Daily fluid balances, once patients were stratified according to the respiratory support, were significantly lower in those requiring mechanical ventilation (*p* = 0.003). No correlation was found between all examined fluid balances and respiratory support duration or oxygen saturation, even after subgroup analysis of patients with invasive mechanical ventilation, or respiratory comorbidities, or bacterial coinfection, or of patients under 1 year old.

**Conclusions:**

In a cohort of children with bronchiolitis, fluid balance was not associated with duration of respiratory support or other parameters of pulmonary function.

**Supplementary Information:**

The online version contains supplementary material available at 10.1186/s44158-023-00093-8.

## Background

Positive fluid balance has been shown to impact respiratory function, with fluid overload (FO) > 10–20% negatively affecting alveolar gas exchange, especially in patients with lung inflammation and in pediatric acute respiratory distress (PARDS) [1–11]. Bronchiolitis is an acute lower respiratory tract disease that affects the distal airways and is typically driven by a viral infection [12]. These infections show a typical epidemiological pattern and are most frequently seen in previously healthy children, generally between 1 and 2 years old, in autumn and winter. The eventual respiratory insufficiency may be mild to severe, and it is mainly obstructive with a restrictive component but without primary involvement of the alveolar epithelium [12–15]. More complex cases may be complicated by secondary consolidation, bacterial coinfections, and pneumonia. These children may be admitted to the pediatric intensive care unit (PICU) for respiratory support [16]. They may be assisted with supplemental oxygen (i.e., high-flow nasal cannula-HFNC-) or require continuous positive pressure ventilation (CPAP), non-invasive ventilation (NIV), or intubation, depending on the severity of respiratory failure [16]. These patients frequently require fluid management because they present at the emergency department with various degrees of dehydration secondary to difficulty in feeding [14]. However, after PICU admission, fluid resuscitation may be required to treat hypovolemia or hypotension caused by positive pressure ventilation or sedation. There is conflicting evidence on the effects of fluid balance on the outcome of pediatric patients affected by viral respiratory diseases [17].

The aim of this study was to explore patterns of daily fluid balances in a cohort of children with bronchiolitis admitted to our PICU. We also assessed the association of daily fluid balance, cumulative FO, and peak FO in the first 7 days of PICU admission with ventilatory support duration and respiratory function. The characteristics of fluid balances were also compared in two different time periods, before and after the severe acute respiratory syndrome coronavirus 2 (SARS-CoV2) pandemic.

## Materials and methods

### Study design and setting

An observational cohort study was conducted including patients with bronchiolitis admitted to Meyer Children’s Hospital PICU from September 2019 to January 2022. We included all patients under 4 years of age with a diagnosis of acute lower respiratory tract viral disease. We excluded patients whose primary admission diagnosis was not due to a respiratory condition and those who were receiving chronic ventilatory support (i.e., at home) before PICU admission. The decision to extend the generally accepted age for bronchiolitis (i.e., 2 years) was taken owing to the unusual wave of older children admitted in 2021–2022 with clear clinical symptoms of bronchiolitis and nasal swab viral positivity [18]. The reporting of this cohort study conforms to the Strengthening the Reporting of Observational Studies in Epidemiology statement [19].

### Data collection

Demographic information, significant clinical history, admission bronchiolitis severity score (BSS), and pediatric index of mortality 3 (PIM3) were gathered from the electronic clinical chart. Clinical data were collected in the first 7 days of PICU admission: the worst daily vital parameters (i.e., heart rate, respiratory rate, systolic, diastolic, systolic/diastolic blood pressure, pulse oxygen saturation (SpO_2_), inspiratory oxygen fraction (FiO_2_)), the worst daily blood gas analysis results (when available), morning laboratory data (i.e., procalcitonin, C-reactive protein, creatinine), type of ventilatory support (i.e., no support, HFNC, CPAP, NIV or invasive mechanical ventilation), including fraction of inspired oxygen. For those who required ventilatory support for more than 7 days, the last day of ventilatory support was recorded. Duration of respiratory support was registered as the number of days when any support was needed, regardless of the modality. In all cases, the length of PICU stay was registered. Patients were categorized according to the most invasive support they had received during the first week of PICU admission. Isolated viruses were reported for all patients, and further pathogens were reported if coinfections occurred.

We calculated fluid balances for all patients (measures are expressed per kg of body weight on PICU admission): all fluid intakes (enteral and parenteral nutrition, blood products, administered medications, and maintenance fluids) and fluid outputs (urine, blood loss, blood sample collections, nasogastric tube drainage, feces, and insensible losses according to patient’s age and ventilation mode) were considered for daily fluid balances. Insensible losses were calculated as 1 ml/kg/h in patients below 10 kg and 0.5 ml/kg/h in others. The cumulative fluid balance was calculated as the sum of daily fluid balances over the first 7 days of PICU admission. Cumulative FO was calculated as the percentage of cumulative fluid balance indexed over admission body weight, and peak FO% as the highest positive fluid balance reached during the first 7 days of stay in PICU, indexed over admission body weight. Diuretics were administered as needed, typically as single boluses, when the clinicians identified a reduced diuresis or an excessively positive fluid balance. This kind of prescription was not standardized.

The indication and timing for PICU admission in our unit are based on a combined assessment of BSS (in case of a score above 4), and clinical evaluation (including respiratory pattern, patient distress, and progression of respiratory insufficiency, patients’ neurologic status). Once in PICU, respiratory support was escalated from HFNC (2 l/min/kg and FiO2 0.3–0.5) to helmet CPAP (positive end-expiratory pressure (PEEP) 6–10, FiO_2_ 0.3–0.6) or full-face mask NIV (pressure support 8–12, PEEP 6–10, FiO2 0.3–0.6), and eventually intubation if clinical signs and symptoms (mainly oxygen saturation and respiratory pattern) did not show improvement. Patients could also be centralized from peripheral hospitals, and in such cases, transportation was generally managed with invasive ventilation (hence, patients were admitted with intubation from the first analyzed PICU day). In these cases, intubation occurred a few hours before transportation to our unit.

The primary objective of this study was to assess daily fluid balances in children with bronchiolitis admitted to PICU. Secondary analyses included the association of daily fluid balances, cumulative FO, and peak FO with respiratory support duration. The association of daily fluid balances with worst daily SpO_2_ was also analyzed. Furthermore, we compared patients admitted before and after the SARS-CoV2 pandemic, who apparently had different disease severity, in order to assess if any difference existed in the two populations in terms of fluid balance and associations with respiratory support duration.

### Statistical analysis

All data are presented as median (interquartile range). Mann–Whitney test was used to assess the differences between variables. Pearson correlation was applied to verify the association between continuous variables. One-way analysis of variance, two-way analysis of variance, and mixed-effects analysis were applied to analyze the differences over time between continuous variables. Tukey’s multiple comparisons post hoc test was applied to assess differences between groups at single time points. A *p* value of < 0.05 was considered statistically significant. Statistical analysis was performed with the GraphPad Prism 9.0 software package (GraphPad Software, San Diego, CA).

## Results

Overall, 103 patients with lower respiratory tract viral infection were admitted to the PICU in the selected periods. Of these, 6 patients were excluded as they were over 4 years old, and in 3 cases respiratory insufficiency was not the primary PICU admission diagnosis. Ultimately, 94 children were admitted in 2 different time periods: 29 between September 2019 and February 2021 and 65 between September 2021 and February 2022.

Median weight, age, isolated pathogens, administration of diuretic therapy, and severity scores are depicted in Table 1.

**Table 1:** Baseline and demographic characteristics of the studied population

**Demographic/baseline**	**Median (IQR)/ [*n*.]**
**Patients *n***	[94]
**Age (months)**	6.9 (1.9–18)
**Weight (kg)**	7.5 (5.3–10)
**PIM3**	0.003 (0.002–0.005)
**BSS**	7 (4–12)
**Baseline HR (bpm)**	140 (120–157)
**Baseline SAP/** **DAP (mmHg)**	107 (94–119)/ 61 (54–71)
**Baseline RR (b/min)**	54 (43–67)
**Baseline SpO2%**	93 (91–95)
**Baseline FiO2**	0.45 (0.35–0.55)
**Received furosemide**^**a**^** (y/n)**	[13/81]
**Isolated pathogen (primary)**	RSV [81], Bocavirus [7], Rhinovirus [2], no isol [4]
**Coinfection (secondary)**	Bocavirus [8], Rhinovirus [1], bacterial [23]

*PIM3* pediatric index of mortality 3, *BSS* bronchiolitis severity score, *HR* heart rate, *SAP* systolic arterial pressure, *DAP* diastolic arterial pressure, *RR* respiratory rate, *SpO2* pulse oxygen saturation, *FiO2* oxygen inspired fraction, *RSV* respiratory syncytial virus

^a^At least a diuretic bolus throughout the PICU admission

In regard to the highest level of respiratory support during the PICU stay, 9 patients received HFNC, 59 children were assisted by CPAP with helmet or by NIV with mask, and in 26 cases mechanical ventilation was needed (3 also required high-frequency oscillatory ventilation).

Respiratory (respiratory rate, FiO_2_, SpO_2_), hemodynamic (heart rate and mean arterial pressure), laboratory and urine output parameters at admission, and their variation over time are described in Supplementary Table S1. Median respiratory support duration was 4 (2–7) days, and it correlated with the PaO2/FIO2 ratio on PICU admission (*r* − 0.5, *p* = 0.002). The median duration of PICU stay was 6 (4–10) days, and hospital stay was 11 (8–17) days. No patient died.

**Fig. 1:**
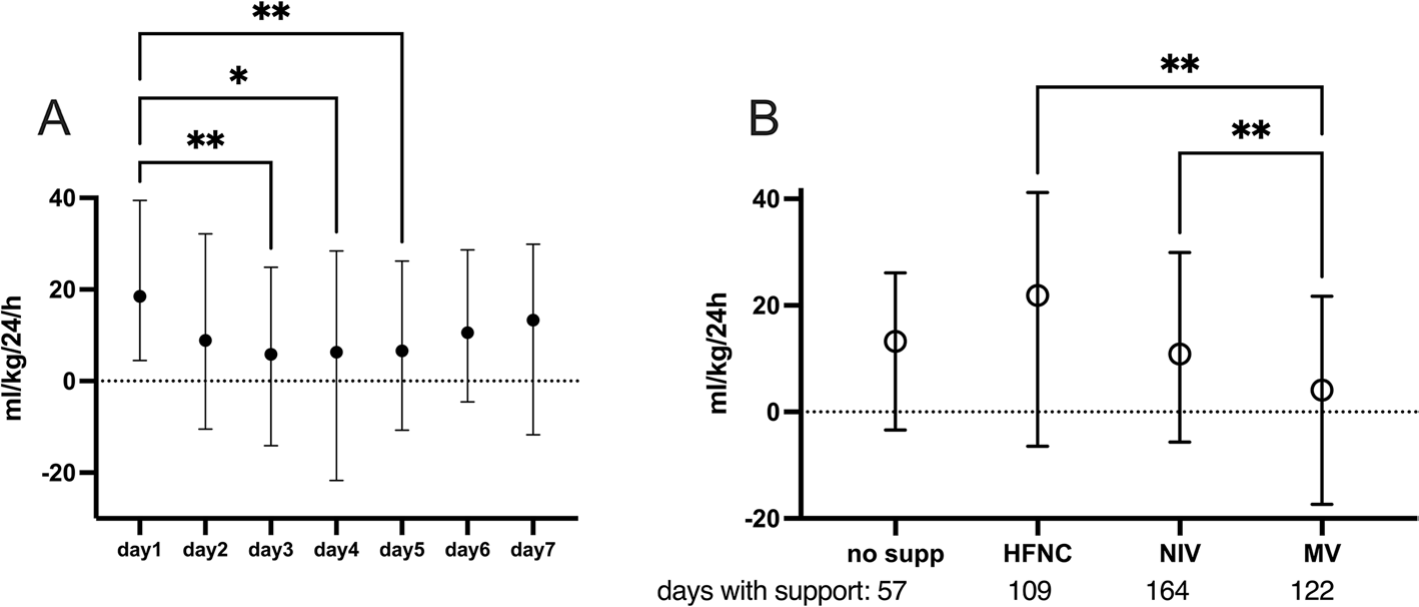
**A** Daily fluid balances from day 1 to day 7 after Pediatric Intensive Care Unit admission showed to change significantly (*p* = 0.0014). At post hoc analysis, they showed significant differences between day 1 and days 2, 3, 4, and 5 respectively. **B** Daily fluid balances compared between different ventilatory modes: days spent with high flow nasal cannula (HFNC) and with non-invasive ventilation (NIV) including face mask and helmet, appeared to have significantly higher fluid balances with respect to those with mechanical ventilation (MV). Data are expressed as median and interquartile range. *Represents *p* < 0.01 and ** represents *p* < 0.001

Median daily fluid balance in the overall cohort was 18 (4.5–19.5) ml/kg at day 1; it decreased to 5.9 (− 14–24.9) ml/kg on day 3 and increased thereafter to 13 (− 11–29.9) ml/kg on day 7 (*p* = 0.001) (Fig. 1A). Cumulative FO% was 4.6 (− 0.8–11) and the median peak FO% was 5.7 (1.9–12.4). Peak FO% was negative or 0 in 18 patients, < 5% in 24, between 5 and 10% in 19, and > 10% in 33.

Daily fluid balances, once patients were stratified according to the respiratory support, were significantly lower in those requiring mechanical ventilation (*p* = 0.003) (Fig. 1B).

We did not find any correlation between respiratory support duration and daily fluid balances, cumulative FO, and peak FO (Table 2). The median length of respiratory support was 3 (1.5.4.5) days in negative or 0 peak FO patients, 4 (2–7) days in those with a FO < 5%, 4 (2.5–6.5) days in children with a FO between 5 and 10%, and 4 (2.2–7) days in patients with a FO > 10%, respectively (*p* = 0.27 at mixed effect analysis).

**Table 2:** Correlation of respiratory support time with daily fluid balance (prokg) from day 1 to day 7, cumulative fluid overload (FO), peak FO, cumulative fluid balances (cumul) from day 1 to day 7. Correlation of the worst pulse oxygen saturation (SpO2) recorded daily from day 1 to day 7 (d1–d7) with daily fluid balance (prokg) from day 1 to day 7

**Respiratory support time vs**			
	*r*	95% CI	*p*
**Prokg d1**	− 0.073	− 0.27 to 0.13	0.49
**Prokg d2**	0.005	− 0.20 to 0.21	0.95
**Prokg d3**	0.102	− 0.12 to 0.31	0.36
**Prokg d4**	− 0.125	− 0.34 to 0.11	0.29
**Prokg d5**	0.013	− 0.25 to 0.27	0.92
**Prokg d6**	− 0.019	− 0.33 to 0.30	0.90
**Prokg d7**	0.203	− 0.14 to 0.50	0.24
**Cumulative FO**	0.010	− 0.19 to 0.21	0.92
**Peak FO**	0.044	− 0.16 to 0.24	0.67
**cumul d1**	− 0.057	− 0.25 to 0.14	0.58
**cumul d2**	0.004	− 0.19 to 0.20	0.96
**cumul d3**	− 0.032	− 0.23 to 0.17	0.75
**cumul d4**	− 0.026	− 0.22 to 0.17	0.80
**cumul d5**	− 0.013	− 0.21 to 0.19	0.89
**cumul d6**	0.010	− 0.19 to 0.21	0.92
**Worst SpO2 (d1–d7) vs**			
	*r*	95% CI	*p*
**Prokg d1**	0.153	− 0.06 to 0.35	0.15
**Prokg d2**	0.129	− 0.08 to 0.33	0.23
**Prokg d3**	0.114	− 0.10 to 0.32	0.30
**Prokg d4**	0.089	− 0.14 to 0.31	0.45
**Prokg d5**	− 0.146	− 0.40 to 0.12	0.29
**Prokg d6**	− 0.113	− 0.42 to 0.22	0.51
**Prokg d7**	0.215	− 0.14 to 0.52	0.23

Similarly, oxygen saturation was not associated in the first 7 days with daily fluid balances (Table 2).

In subgroup analysis, after inclusion of patients requiring invasive mechanical ventilation, or patients with respiratory comorbidities, or patients with bacterial coinfection, or patients under 1 year old, we still could not find any correlation between fluid balance and respiratory support duration (Supplementary Table S2).

The analysis of patients admitted to the PICU between 2019 and 2021, compared to those admitted between 2021 and 2022, showed that in the timeframe from September 2020 to February 2021 no patient with bronchiolitis was admitted. Hence, essentially equal periods (i.e., September 2019–February 2020 vs. September 2021–February 2022) were compared and about a double number of patients were admitted in the second period. Fluid balances over time were significantly lower in the first era (*p* = 0.0002), as well as weights (*p* = 0.03) and ages (*p* = 0.01), whereas PIM3 was higher (*p* = 0.001) (Table 3). However, respiratory support durations (*p* = 0.72) were not different in the two periods and their correlation with daily fluid balances was absent in both eras (all *p* > 0.05) (Table 3).

**Table 3:** Comparison of the main characteristics pre- and post-pandemic patients with signs of viral lower respiratory tract infection

	**2021–2022**		**2019–2021**	
**Comparisons**				*p*
**Respiratory support time (days)**	4 (2–6.5)		4 (2–7)	0.72
**PIM3**	0.002 (0.001–0.004)		0.005 (0.004–0.005)	0.001
**Age (months)**	8.1 (2.6–22)		2.9 (1.5–9.8)	0.01
**Weight (kg)**	8.6 (5.5–11)		6 (4.4–8.5)	0.03
**Correlations**				
**Respiratory support time vs**	*r*	*p*	*r*	*p*
**Prokg d1**	− 0.05	0.70	0.29	0.13
**Prokg d2**	− 0.09	0.50	0.19	0.35
**Prokg d3**	0.12	0.36	0.05	0.80
**Prokg d4**	0.00	0.99	− 0.07	0.74
**Prokg d5**	0.07	0.60	− 0.27	0.27
**Prokg d6**	0.24	0.05	0.02	0.95
**Prokg d7**	0.01	0.95	0.13	0.68

*PIM3* pediatric index of mortality 3. The table also reports the correlation of respiratory support time with daily fluid balances (prokg) from day 1 to day 7 in both groups

## Discussion

According to our results, the duration of respiratory support and oxygen saturation in patients with a diagnosis of lower respiratory tract viral infection do not seem to be correlated with fluid balance.

Daily fluid balances in our patients showed a U-shape owing to fluid resuscitation during the first 24–48 h, eventually followed by fluid restriction and increased diuresis in days 3 and 4 of their PICU stay, according to the recommendations on fluid management for respiratory pediatric patients [20]. Fluid balance increased thereafter, probably as a result of enteral nutrition and liberal fluid administration during the recovery phase. Many studies have demonstrated the negative role of fluid accumulation in both adult and pediatric patients with multiorgan dysfunction [21, 22]. However, a similar U-shaped trajectory of daily fluid balances during the first 7 days after PICU admission is shown in the study by Flores-Gonzalez et al. [23], who focused on fluid balance in infants with bronchiolitis and enrolled a heterogenous cohort very similar to ours. These authors found that only positive fluid balance 24 h after PICU admission was associated with hospital length of stay and duration of invasive and non-invasive mechanical ventilation (but not thereafter), and it was not associated with the need for intubation. The impact of fluid balance on blood oxygenation was not analyzed. Ingelse et al. [24] showed a cumulative positive fluid balance of 150 mL/kg at day 7 (that corresponds to a FO of 15%). They included only intubated patients and found a correlation between cumulative fluid balance at day 3 and duration of mechanical ventilation, but they were unable to find any association with blood oxygenation. In our opinion, these studies report inconclusive results, and no clear indication on which day after PICU admission has an association with outcomes, in terms of fluid balance. Furthermore, they did not identify a clear pathophysiological association between daily fluid balance and pulmonary function throughout the first 7 days of PICU admission.

Compared with these studies and others [23–26], however, we performed the most accurate evaluation of median daily fluid balance, cumulative FO, and peak FO. Even if these data are difficult to compare, given the absence of a standardization for fluid balance assessment and analysis, our cohort seemed to have less positive fluid balances with respect to previous studies. Moreover, our intubated critically ill patients seemed to receive the lowest amount of fluids and reached a more negative fluid balance. These aspects may explain the lack of association between fluid balance and respiratory outcomes in our patients. However, even if about half of our patients reached a negative fluid balance by day 3 and the median level of peak FO was 5–6%, we recorded a maximal peak FO of about 30% in some patients. Furthermore, 1 patient over 3 in our cohort showed to reach a peak FO% above 10%. Recently, it has been noted that in critically ill children it can be very difficult to set a specific threshold of harmful FO [22], even if there might be no signs of harm in mechanically ventilated patients up to a threshold of 10%. In essence, in our study, we did not find any breakpoint in the association of fluid balance with the number of days of respiratory support.

We decided to compare the pre- and post-pandemic eras since we noticed some differences in the infections rate and severity. In fact, after the SARS-CoV2 pandemic, older patients (i.e., ranging from 1 month to 4 years old) have been admitted with lower respiratory tract viral infection compared to the previous period. The number of these admissions to the PICU was two-fold in 2021–2022 with respect to previous years. This could be due to the “immunity debt” caused by the strict non-pharmacologic interventions against SARS-CoV2, which led to a substantial reduction in typical seasonal viruses among infants and children, who did not develop any kind of immunity against these pathogens [18, 27–32]. Interestingly, we confirmed that severity scores were significantly different between the pre- and post-pandemic groups. We also observed a different fluid management with an apparently more restrictive approach in the pre-pandemic era. This finding is not easy to interpret based on the present data. It is possible that our approach has become more liberal after the pandemic in patients with bronchiolitis. However, we showed that the impact of fluid balance on lung function and ventilation was not different between the two waves of viral lower respiratory tract infections, which suggests that the pathophysiology did not change in these two periods. Interestingly, the different fluid managements did not seem to affect the (respiratory) outcomes and this possibly reinforces the initial hypothesis.

### Limitations

Our retrospective study has several limitations. We did not attempt to associate the duration of respiratory support with PaO_2_/FiO_2_ because of several missing data (blood gas analyses were not available for all patients every day). The lack of association between fluid balances and respiratory support duration in our study may be due to a low signal, since median FO and median peak FO were below the “recognized” threshold of danger. However, our results may imply that managing fluids within such threshold is safe. Moreover, even the subgroup of patients with the highest positive fluid balances (i.e., above 10%) did not show worse outcomes. In other words, our study might contribute to show that in children with bronchiolitis this threshold (10%) does not affect lung function. Respiratory support duration (including all support modes) may be a suboptimal outcome and can be influenced by attending clinicians’ personal evaluations. However, we estimated that PICU and hospital stay were also influenced by many factors, including logistical requirements (i.e., anticipated or delayed transferal from PICU to ward or hospital discharge owing to unpredictable availability of beds). Fluid balance before PICU and hospital admission could not be assessed. This issue should be more deeply investigated, especially in infants, as bronchiolitis could be associated with difficulty in feeding and with dehydration. Fluid administration beyond maintenance fluids did not follow any goal-directed protocol but was administered to optimize suspected hypovolemia (fluid challenge boluses of 10 ml/kg). This aspect may significantly affect the amount of input fluids. As a matter of fact, our patients were generally hemodynamically stable with healthy cardiac function and prompt response to fluid boluses. For this reason, our center tended to apply a relatively restrictive fluid management. How to standardize the calculation of inputs and outputs remains unclear. In accordance with Ingelse et al. and Flores-Gonzalez et al., in our study the contribution of enteral feeding to daily input was considered exactly as parenteral input [23, 24], which may not be correct. On the other hand, the calculation of insensible losses is not considered by all authors (i.e., by Ingelse et al.) but we included it in our balances. Furthermore, it is currently uncertain which insensible loss formula should be adopted. In fact, conditioned air, in PICU boxes, and humidified gasses, applied in 100% of these patients, may be considered as a compensation for insensible losses [33]. Fluid balance may have impacted respiratory support in patients with difficult ventilation and with “aggressive” ventilator parameters and we did not verify this aspect in the present study. However, our cohort, representative of an “average” population of children with severe bronchiolitis, rarely required aggressive ventilation.

## Conclusions

In a cohort of children with bronchiolitis admitted to PICU for respiratory insufficiency, fluid balance was positive at day 1, it decreased on day 3 and increased thereafter with a median peak FO of about 5%. Fluid balance was not associated with respiratory support duration or other parameters of pulmonary function regardless of age, type of respiratory support, comorbidities, or the presence of bacterial coinfection. Further standardization of fluid balance assessment is needed to ultimately clarify whether fluid management has an impact on outcomes of children with lower respiratory tract viral infection.

## Supplementary Information

**Additional file 1: Supplementary Table S1.** Laboratory, hemodynamic, respiratory variables in the studied population from day 0 to day 7.** Supplementary Table S2.** Correlation of different subgroups with fluid balance from day 1 to day 7.

## Data Availability

The datasets used and/or analyzed during the current study are available from the corresponding author on reasonable request.

## Abbreviations

FO: Fluid overload
PARDS: Pediatric acute respiratory distress
PICU: Pediatric intensive care unit
HFNC: High-flow nasal cannula
CPAP: Continuous positive pressure ventilation
NIV: Non-invasive ventilation
SARS-CoV2: Severe acute respiratory syndrome coronavirus 2
BSS: Bronchiolitis severity score
PIM3: Pediatric index of mortality 3
SpO_2_: Pulse oxygen saturation
FiO_2_: Inspiratory oxygen fraction
PEEP: Positive end-expiratory pressure
