# Supplementary material for: Fluid balance in critically ill children with lower respiratory tract viral infection: a cohort study
Source: J Anesth Analg Crit Care. 2023 Apr 28;3:10. doi: 10.1186/s44158-023-00093-8 (PMC10141805; doi:10.1186/s44158-023-00093-8)
Supplement: Supplementary file 1 — Additional file 1: Supplementary Table S1. Laboratory, hemodynamic, respiratory variables in the studied population from day 0 to day 7. Supplementary Table S2. Correlation of different subgroups with fluid balance from day 1 to day 7. [file 44158_2023_93_MOESM1_ESM.docx]

Supplementary Table 1. Laboratory, hemodynamic, respiratory variables in the studied population from day 0 to day 7.

|  | **day 0** | **day 1** | **day 2** | **day 3** | **day 4** | **day 5** | **day 6** | **day 7** |
| --- | --- | --- | --- | --- | --- | --- | --- | --- |
| **sCr (mg/dl)** | 0.22 (0.17-0.26) | 0.25 (0.17-0.28) | 0.26 (0.21-0.32) | 0.21 (0.18-0.3) | 0.33 (0.23-0.34) | 0.36 (0.25-0.58) | 0.42 (0.2-0.7) | 0.26 (0.15-0.37) |
| **CRP (mg/dl)** | 0.6 (0.14-3) | 0.88 (0.29-2.9) | 1.89 (0.33-2.90) | 1.21 (0.15-5.0) | 2.8 (0.3-8.3) | 1.81 (0.4-5.5) | 0.3 (0.1-6.9) | 0.86 (0.10-5.8) |
| **PCT (mg/dl)** | 0.19 (0.1-1.5) | 0.15 (0.1-1) | 0.46 (0.1-1.7) | 0.1 (0.07-1.35) | 0.71 (0.19-1-1) | 0.56 (0.1-1.6) | 0.11 (0.5-0.4) | 0.1 (0.09-0.35) |
| **UOP (ml/kg/day)** | - | 47 (20-73) | 78 (53-104) | 83 (61-111) | 77 (59-109) | 72 (57-118) | 88 (45-113) | 84 (59-110) |
| **SAP mmHg** | 107 (94-119) | 103 (92-120) | 102 (88-108) | 100 (90-109) | 100 (90-108) | 96 (84-111) | 97 (86-109) | 99 (85-109) |
| **DAP (mmHg)** | 61 (54-71) | 58 (47-66) | 55 (46-65) | 54 (44-66) | 52 (44-67) | 54 (46-68) | 54 (42-69) | 55 (46-64) |
| **Heart rate (beat/m)** | 140 (123-157) | 128 (111-145) | 117 (102-140) | 127 (102-144) | 118 (102-139) | 128 (105-141) | 123 (109-138) | 123 (104-141) |
| **Resp rate (breath/m)** | 54 (44-66) | 45 (34-58) | 40 (32-50) | 41 (31-50) | 40 (32-57) | 38 (32-48) | 40 (32-61) | 40 (30-52) |
| **SpO2 %** | 93 (91-95) | 93 (91-95) | 94 (92-96) | 94 (92-96) | 94 (92-95) | 94 (93-95) | 95 (92-96) | 95 (92-96) |

Supplementary Table 2. Correlation of different subgroups with fluid balance from day 1 to day 7.

| **bacterial** | **Day 1** | **Day 2** | **Day 3** | **Day 4** | **Day 5** | **Day 6** | **Day 7** |
| --- | --- | --- | --- | --- | --- | --- | --- |
| r | -0.23 | 0.12 | 0.007 | -0.15 | -0.35 | 0.06 | -0.08 |
| 95% confidence interval | -0.58 to 0.20 | -0.32 to 0.52 | -0.43 to 0.43 | -0.56 to 0.32 | -0.73 to 0.18 | -0.52 to 0.61 | -0.62 to 0.51 |
| p | 0.29 | 0.59 | 0.99 | 0.52 | 0.18 | 0.84 | 0.8 |
| **infants** |  |  |  |  |  |  |  |
| r | 0.026 | 0.052 | 0.055 | -0.22 | -0.46 | -0.076 | 0.048 |
| 95% confidence interval | -0.23 to 0.28 | -0.21 to 0.31 | -0.22 to 0.32 | -0.48 to 0.08 | -0.69 to -0.16 | -0.46 to 0.32 | -0.37 to 0.45 |
| p | 0.84 | 0.70 | 0.69 | 0.14 | 0.00 | 0.71 | 0.82 |
| **respiratory** |  |  |  |  |  |  |  |
| r | 0.22 | -0.13 | 0.14 | 0.085 | -0.025 | 0.5312 | -0.60 |
| 95% confidence interval | -0.28 to 0.63 | -0.55 to 0.34 | -0.33 to 0.56 | -0.41 to 0.54 | -0.56 to 0.53 | -0.2773 to 0.8993 | -0.91 to 0.17 |
| p | 0.38 | 0.59 | 0.55 | 0.74 | 0.93 | 0.1755 | 0.11 |
| **intubated** |  |  |  |  |  |  |  |
| r | -0.11 | 0.13 | 0.27 | -0.17 | -0.11 | 0.04816 | 0.19 |
| 95% confidence interval | -0.49 to 0.30 | -0.28 to 0.51 | -0.13 to 0.60 | -0.55 to 0.26 | -0.53 to 0.34 | -0.4283 to 0.5037 | -0.31 to 0.62 |
| p | 0.59 | 0.53 | 0.19 | 0.42 | 0.62 | 0.8495 | 0.44 |
